# PARP10 (ARTD10) modulates mitochondrial function

**DOI:** 10.1371/journal.pone.0187789

**Published:** 2018-01-02

**Authors:** Judit Márton, Tamás Fodor, Lilla Nagy, András Vida, Gréta Kis, Attila Brunyánszki, Miklós Antal, Bernhard Lüscher, Péter Bai

**Affiliations:** 1 Department of Medical Chemistry, Faculty of Medicine, University of Debrecen, Debrecen, Hungary; 2 MTA-DE Cell Biology and Signaling Research Group, Debrecen, Hungary; 3 MTA-DE Lendület Laboratory of Cellular Metabolism, Debrecen, Hungary; 4 Department of Anatomy, Histology and Embryology, Faculty of Medicine, University of Debrecen, Debrecen, Hungary; 5 MTA-DE Neuroscience Research Group, Debrecen, Hungary; 6 Institute of Biochemistry and Molecular Biology, Medical School, RWTH Aachen University, Aachen, Germany; 7 Research Center for Molecular Medicine, University of Debrecen, Debrecen, Hungary; University of South Alabama, UNITED STATES

## Abstract

Poly(ADP-ribose) polymerase (PARP)10 is a PARP family member that performs mono-ADP-ribosylation of target proteins. Recent studies have linked PARP10 to metabolic processes and metabolic regulators that prompted us to assess whether PARP10 influences mitochondrial oxidative metabolism. The depletion of PARP10 by specific shRNAs increased mitochondrial oxidative capacity in cellular models of breast, cervical, colorectal and exocrine pancreas cancer. Upon silencing of PARP10, mitochondrial superoxide production decreased in line with increased expression of antioxidant genes pointing out lower oxidative stress upon PARP10 silencing. Improved mitochondrial oxidative capacity coincided with increased AMPK activation. The silencing of PARP10 in MCF7 and CaCo2 cells decreased the proliferation rate that correlated with increased expression of anti-Warburg enzymes (Foxo1, PGC-1α, IDH2 and fumarase). By analyzing an online database we showed that lower PARP10 expression increases survival in gastric cancer. Furthermore, PARP10 expression decreased upon fasting, a condition that is characterized by increases in mitochondrial biogenesis. Finally, lower PARP10 expression is associated with increased fatty acid oxidation.

## Introduction

Poly(ADP-ribosyl)ation (PARylation) is an evolutionarily conserved biochemical reaction that involves the catalytic cleavage of NAD^+^ to nicotinamide and ADP-ribose and the subsequent addition of the ADP-ribose (ADPR) moiet(ies) to acceptor proteins [1]. Poly(ADP-ribose) polymerase (PARPs/ARTDs) are the enzymes responsible for cellular PARylation. “Classical” PARP enzymes (e.g. PARP1, PARP2) cleave NAD^+^, then transfer ADPR to target proteins in an iterative process that results in the synthesis of protein-bound ADPR polymers (PAR). Not all PARPs are capable of PARylation. Family members that lack a catalytic glutamate are unable to perform this iterative transfer of ADPR units, hence, they use a mechanism called substrate assisted catalysis. These enzymes, including PARP10/ARTD10, only mono-ADP-ribosylate their substrates [2]. PARPs are involved in numerous cellular processes, among them metabolic regulation (for review see [3]). PARP1-evoked mitochondrial damage was described in 1998 [4], in light of recent investigations it seems that PAR metabolism has a rather complex regulatory role on mitochondrial oxidative capacity [5]. To date the expression and activity of PARP1, PARP2 showed characteristic inverse correlation with mitochondrial activity [5].

Mitochondria are intracellular organelles that are responsible for a wide array of biochemical processes, however, mitochondria are best known for harboring biological oxidation. Mitochondrial oxidative capacity is a very efficient way to generate ATP in cells. Therefore, in tissues with a high energy requirement mitochondrial oxidative capacity has a pivotal role in providing ATP, and as a logical continuation, mitochondrial damage is deleterious in these tissues [6]. Chronic decrease in mitochondrial oxidative capacity correlates with metabolic diseases (e.g. type II diabetes), with aging and with cancer development [6–8]. Acute changes in mitochondrial activity occur in response to nutrient availability, for example during fasting mitochondrial activity is enhanced [9].

PARP10 (ARTD10) is multidomain protein that performs mono-ADP-ribosylation of target proteins [2]. PARP10 activity seems to be regulated by posttranslational modifications (for comprehensive review see [10]). In quiescent, non-stressed cells the majority of PARP10 is cytosolic due to a strong nuclear export signal [11]. Both nuclear (e.g. c-MYC, RAN, and histones) and cytosolic (e.g. GSK3β, NEMO/IKKγ, and PCNA) substrates of PARP10 have been identified [11–16]. Importantly, PARP10 seems to be involved in metabolic regulation. PARP10 mono-ADP-ribosylates glycogen synthase kinase 3β (GSK3β) [12] and interacts with c-MYC [11]. Both GSK3β and c-MYC are involved in various metabolism-related signaling events [17]. Furthermore, genome wide association and proteome studies linked PARP10 to lipid metabolism [18, 19]. These preliminary data prompted us to assess a potential role of PARP10 in mitochondria-associated metabolic activities.

## Methods

### Chemicals

Chemicals were from Sigma-Aldrich (Budapest, Hungary) if not stated otherwise.

### Cell culture

MCF-7, HeLa, CaCo2 and Capan3 cells were maintained in MEM (Sigma-Aldrich, Budapest, Hungary), 10% fetal bovine serum (Sigma-Aldrich), 1% Penicillin/Streptomycin (Invitrogen, Carlsbad, California, USA), 2 mM L-Glutamine in humidified atmosphere 95% air-5% CO_2_ at 37°C. Cells were serum or glucose-fasted for 12 hours where indicated.

The cell lines used in the study are available from European Collection of Authenticated Cell Cultures (ECACC).

### Constructs, transfections

In PARP10 silencing experiments the pSUPER RNAi system was used [20]. Constructs expressing shRNAs specific for PARP10 have been described in [12]; in the experiments shown in the current manuscript a mixture of two PARP10 shRNA constructs were used. Transfections were performed on two consequtive days using Jet-PEI transfection reagent (Polyplus Transfection, New York, USA).

### Assessment of cellular proliferation

For assessing cell proliferation we applied the sulphoradamine B (SRB) assay, or, as an alternative, BRDU-incorporation assay using the Cell Proliferation ELISA, Brdu (colorimetric) kit (Hoffmann-La Roche, Basel, Switzerland) according to the manufacturer’s instructions. In the SRB assay cells were seeded in 96-well plate (3000–5000 cells/well) and depleted of PARP10 using specific shRNA expressing constructs. At the end of the treatment cells were fixed *in situ* by 50% trichloroacetic acid (TCA) and subsequently stained with sulforhodamine B (SRB) solution (0.4% in 1% acetic acid). Unbound dye was removed by washing with 1% acetic acid. Bound stain was solubilized in 10 mM TRIS base. Absorbance was read on an automated plate reader (Thermo Labsystems Multiskan MS, Walthman, Massachusetts, USA) at 540 nm.

### Measurement of superoxide production

Superoxide was measured using hydroethidine (HE) staining. Cells were seeded in 96-well plate (25 000 cells/well). PARP10 was depleted for 2 days, then cells were stained with 2 μM DioC6 for 30 min and subsequently harvested by trypsin/EDTA. Cells were then subjected to flow cytometric analysis (FACSCalibur, BD Biosciences, Mountain View, CA) with 20,000 events collected for each sample.

### Oxygen consumption

Oxygen consumption was measured using an XF96 oxymeter (Seahorse Biosciences, North Billerica, MA, USA) similarly to a previously published protocol [21]. In brief, cells were seeded in 96-well assay plates (~2000 cell/well) and PARP10 was depleted by shRNA transfection. Oxygen consumption rate (OCR, reflecting mitochondrial oxidative capacity) and changes in pH, termed extracellular acidification rate (ECAR, reflecting glycolysis) were recorded. Cells were treated with etomoxir (50 μM), then oligomycin (10 μM) and finally antimycin (10 μM). Data were normalized to protein content and normalized readings were used for calculations. OCR values after antimycin treatment were subtracted from all other values. We named the readings for the untreated cells *baseline OCR*. OCR after etomoxir treatment represents the oxygen consumption (mostly) related to glucose and amino acid oxidation (*Glc+AA*), while the difference between baseline and Glc+AA OCR represents fatty acid oxidation (*FAO*). The oligomycin-resistant respiration gives information on the leakage through the inner membrane of the mitochondria (*proton leak*).

### RNA isolation, reverse transcription-coupled QPCR (RT-qPCR)

Total RNA was prepared using the TRIzol reagent (Invitrogen, Carlsbad, California, USA) according to the manufacturer’s instructions. Two micrograms of RNA were used for reverse transcription (High Capacity cDNA Reverse Transcription Kit, Applied Biosystems, Foster City, CA, USA). Diluted cDNA was used for reverse transcription-coupled quantitative PCR (RT-qPCR). The qPCR reactions were performed with the qPCRBIO SyGreen Lo-ROX Supermix (PCR Biosystems, London, UK) using a Light-Cycler 480 system (Roche Applied Science, Basel, Switzerland). Gene expression was normalized to the geometric mean of human 36B4, 18S, and cyclophyllin values. Primers are listed in Table 1.

**Table 1:** List of primers used in the study.

Cyclophilin A (human)	F: GTCTCCTTTGAGCTGTTTGCAGAC
Cyclophilin A (human)	R: CTTGCCACCAGTGCCATTAT
FOXO1 (human)	F: GTTCATTGAGCGCTTAGACTG
FOXO1 (human)	R: AAGTGTAACCTGCTCACTAACCC
Fumarase (human)	F: CTCGTTTTGGCCTCCGAACG
Fumarase (human)	R: TAACTGGGGTTGGCATGCGT
IDH2 (human)	F: GGTGGAGATGGATGGTGATGA
IDH2 (human)	R: GTGATGGTGGCACACTTGACT
PGC-1α (human)	F. AGAATTGGCTTATGGATGTACAGG
PGC-1α (human)	R: TTTGTTGATCATTTCCAGCAATAAT
18s (human)	F: TCGAGGCCCTGTAATTGGAAT
18s (human)	R: TCCCAAGATCCAACTACGAGCTT
36B4 (human)	F: CCATTGAAATCCTGAGTGATGTG
36B4 (human)	R: GTCGAACACCTGCTGGATGAC
PARP10 (human)	F: CTGTGGACCTGCTGTTGCTG
PARP10 (human)	R: GGATGTCGTAGTGGGGGACA
PARP1 (human)	F: CACTGGTACCACTTCTCCTGCTTC
PARP1 (human)	R: CTTTGCCTGTCACTCCTCCAG
PARP2 (human)	F: GCTAAATCAGACCAATCTCC
PARP2 (human)	R: CAGGCTGTGCTGTCCCATTT
PARP3 (human)	F: CTTCCTGGGCCTCATCCTCTG
PARP3 (human)	R: CAACCGCTTCTTCACCTGCTG
PARP4 (human)	F: AAAAGCGCACAGACTGCAAAG
PARP4 (human)	R: CACTGGCTAGGTCAGGGAGGT
PARP5a (human)	F: AACATCCTTCCTTCCAAAACCT
PARP5a (human)	R: GGCAAACGTAAATGCAAAGG
PARP5b (human)	F: AAGGTTACCCGGCAAAAGA
PARP5b (human)	R: TGGGTGTCCAGTTCACAAAG
PARP6 (human)	F: GTCTTGGGATCAGTGGGGTCC
PARP6 (human)	R: CCTTCTACACACTGGGCGTCA
PARP7 (human)	F: TGGACAGCCTTCGTAGTTGGT
PARP7 (human)	R: GGCAGATTTGAATGCCATGA
PARP8 (human)	F: ATGTGAACGGGAGCTGTGTGT
PARP8 (human)	R: TTGAAGGCCAACATCTGAGGA
PARP9 (human)	F: CGGATGTCCCTGGCAGAAGAA
PARP9 (human)	R: ACTCGACACCTTGCGATCCAA
PARP11 (human)	F: AAAGCTTCCTTGACCACCGAGA
PARP11 (human)	R: ACATGCGACCTCCTTCCAAAGA
PARP12 (human)	F: CAGAGGGCCAGGTTCTGTACTC
PARP12 (human)	R: GACCCAAATACGTGTCTCCCCA
PARP14 (human)	F: CACCTGGAAGATGATGGAGCCA
PARP14 (human)	R: GAGGTTCACTTTCTGCTGCACC
PARP15 (human)	F: CAAGAGGACAGTGGGGTAGGTG
PARP15 (human)	R: AGTCTGGGAGGGAATTGCACAA
PARP16 (human)	F: ACCACTGACAGCTTGGACACTT
PARP16 (human)	R: GCCTTTGATGATGTTCCCAGCC
NDUFA2 (human)	F: CGATATTAACAAGGATGGCGG
NDUFA2 (human)	R: TCTCAATGAAGTCCCTGACGC
NDUFA3 (human)	F: AGACAAAGATGGCTGCGAGAG
NDUFA3 (human)	R: CGTGGCCTTGTTGATCATGAC
NDUFB5 (human)	F: GTATTCATTGGTCAAGCTGAACTAG
NDUFB5 (human)	R: CAGCTCCTTTACCCGTAATTCAGC
PGC1-β (human)	F: GTGCTGACAAGAAATAGGAGAGG
PGC1-β (human)	R: CTCTTCTGAATTGGAATCGTAGTC
UCP2 (human)	F: CTACAAGACCATTGCCCGGAG
UCP2 (human)	R: ACAATGGCATTACGAGCAACA
ATP5G1 (human)	F: CTAAACAGCCTTCCTACAGCAACTT
ATP5G1 (human)	R: TGAACCAGCCACACCAACTGT
CYCS (human)	F: TAAGAACAAAGGCATCATCTGG
CYCS (human)	R: AGGCAGTGGCCAATTATTACTC
COX7A1 (human)	F: ATACGGAAACAGGCTCGGAGGT
COX7A1 (human)	R: ATCCGTTTCGGTCTCGGAATTT
COX17 (human)	F: GATGCGTGTATCATCGAGAAAG
COX17 (human)	R: CAGCAGACCACCATTTCATATTT
CPT1A (human)	F: CAGGCGAGAACACGATCTTC
CPT1A (human)	R: GCGGATGTGGTTTCCAAAG
CPT2 (human)	F: CCAGGCTGCCTATTCCCAAAC
CPT2 (human)	R: AGGGTCCCGAAATGTAGCTTG
MCAD (human)	F: AGAATTGGCTTATGGATGTACAGG
MCAD (human)	R: TTTGTTGATCATTTCCAGCAATAAT
ME2 (human)	F: TGTTAAGGCTGTTGTAGTGACTGA
ME2 (human)	R: TAAGAGTGCGATATTATCAGTTCCC
GAPDH (human)	F: CGTATTGGGCGCCTGGTC
GAPDH (human)	R: GGAATTTGCCATGGGTGGAA
MLYCD (human)	F: AACATCCAGGCAATCGTGAAG
MLYCD (human)	R: GAGGAAACTCTCTCTGCAACTCC
PKLR (human)	F: CACCAAGAACCCCAGAACTCC
PKLR (human)	R: GGAATAAGGGAAAGGCCCAAG
CAT (human)	F: TGAAAATTTGTGCATCCTTCA
CAT (human)	R: ATTCTGGAGAAGTGCGGAGA
SOD1 (human)	F: CCCACCGTGTTTTATGGATA
SOD1 (human)	R: AGGTGTGGGGAAGCATTAAA
SOD2 (human)	F: AATCAGGATCCACTGCAAGG
SOD2 (human)	R: TAAGCGTGCTCCCACACAT
SDHB (human)	F: CCTTCGGGTGCAAGCTAGAGT
SDHB (human)	R: CAAGGCTGGAGACAAACCTCA
PARP1 (mouse)	F: GGAGCTGCTCATCTTCACC
PARP1 (mouse)	R: GCAGTGACATCCCCAGTACA
PARP2 (mouse)	F: GGAAGGCGAGTGCTAAATGAA
PARP2 (mouse)	R: AAGGTCTTCACAGAGTCTCGATTG
PARP3 (mouse)	F: CCTGCTGATAATCGGGTCAT
PARP3 (mouse)	R: TTGTTGTTGTTGCCGATGTT
PARP4 (mouse)	F: GTTAAATTTTGCACTCCTGGAGA
PARP4 (mouse)	R: AATGTGAACACTGTCAAGAGGAAC
PARP5a (mouse)	F: TAGAGGCATCGAAAGCTGGT
PARP5a (mouse)	R: CAGGCATTGTGAAGGGG
PARP5b (mouse)	F: GGCCCTGCTTACACCATTG
PARP5b (mouse)	R: CGTGCTTGACCAGAAGTTCA
PARP6 (mouse)	F: TTTCCAGCCATCGAATAAGG
PARP6 (mouse)	R: ACCACTTGCCTTGAACCAAC
PARP7 (mouse)	F: AAAACCCCTGGAAATCAACC
PARP7 (mouse)	R: AGAAGGATGCGCTTCTGGTA
PARP8 (mouse)	F: TCCACCATTAAATCGCACAA
PARP8 (mouse)	R: GCTCCATTTTCGATGTCTTG
PARP9 (mouse)	F: ACCTGAAGAATGGCCTATTACATGG
PARP9 (mouse)	R: ACAGCTCAGGGTAGAGATGC
PARP10 (mouse)	F: CAAGATCCTGCAGATGCAAA
PARP10 (mouse)	R: TTGGAGAAGCACACGTTCTG
PARP11 (mouse)	F: CAATGAGCAGATGCTATTTCATG
PARP11 (mouse)	R: CACCAATTAGCACTCGAGCA
PARP12 (mouse)	F: CGGATCCAVAACATGGGC
PARP12 (mouse)	R: GGCATCTCTCGCAAAGTAGC
PARP14 (mouse)	F: GGCAAACGCAATGGAACTAT
PARP14 (mouse)	R: AGCACGTTCCTAAGCCTTGA
PARP16 (mouse)	F: CCGTGTGCCTTATGGAAACT
PARP16 (mouse)	R: TGGATTGTGTCTGGGCAC

### SDS-PAGE and Western blotting

Cells were lysed in RIPA buffer (50 mM Tris, 150 mM NaCl, 10% SDS, 1% Nonidet P-40, 1 mM Na_3_VO_3_, 1 mM NaF, 0.5% sodium deoxycholate, 1 mM phenylmethylsulfonyl fluoride, protease inhibitor mixture, pH 8.0). Proteins were separated by sodium dodecyl sulfate-polyacrylamide gel electrophoresis (SDS-PAGE) on 8% acrylamide gels and blotted onto nitrocellulose membranes. After blocking in 5% (w/v) non-fat dry milk, the membranes were washed with 1x TW-TBS and incubated with primary antibodies at 4°C overnight. The primary antibodies are listed in Table 2. Blots were quantified by densitometry using the Image J software. Upon evaluation the values of the pSuper-transfected cells were considered 1, hence, the shPARP10 values are fold changes. Due to the lack of variance in the control values we did not perform statistical evaluation. The pAMPK/AMPK ratio was calculated using the average pAMPK and average AMPK values.

**Table 2:** List of the primary antibodies used in the current study.

Target	Company	Dilution
AMPK	Sigma-Alrdich	1:500
phospho-AMPK (pAMPK)	Cell Signaling, Danvers MA, USA	1:1000
PARP10	rat anti ARTD10 Clone 5H11 [16]	1:1000
anti-beta-actin peroxidase-coupled antibody	Sigma-Alrdich	1:20000
PGC-1α	Abcam, Cambridge UK	1:1000

The secondary antibody was IgG peroxidase HRP conjugate (Sigma-Alrdich, 1:2000), anti-rat-POD 1:1000. Bands were visualized by enhanced chemiluminescence reaction (West Pico ECL Kit, Thermo Scientific, Walthman, Massachusetts, USA).

### Electron microscopy

Pellets of cells were processed for electron microscopic investigation. Briefly, cells were fixed in 3% glutaraldehyde dissolved in 0.1 M cacodylate buffer (pH: 7.4) containing 5% sucrose for 1 hour at RT. After washing several times in cacodylate buffer (pH: 7.4), cells were post-fixed in 2% osmium tetroxide for 2 hours at RT. Following several washes in cacodylate buffer (pH: 7.4), cells were dehydrated and embedded into Durcupan ACM resin. Ultrathin sections were cut, collected on Formvar-coated single-slot grids, and counterstained with uranyl acetate and lead citrate. Sections were investigated with a JEOL 1010 transmission electron microscope and photographed at a magnification of 6000-10000x with an Olympus Veleta CCD camera. Digitalized images were processed with Adobe Photoshop CS5 software.

Morphometric assessment was accomplished using the Image J software as follows. The EM pictures of at least 20 different cells of each group were analyzed. Twenty cells in each experimental group were analyzed in single EM micrographs. The numbers of mitochondria were counted. The cross-sectional area of mitochondria and the whole area of the investigated cells were measured. From the measured values the total and the average crossectional areas of mitochondria were calculated. Finally, the proportion of the surface area of the cell covered by the mitochondria was also calculated. From the measured and calculated data obtained from the 20 investigated cells average values and standard errors of means were calculated. Statistical differences among the data sets were calculated by using the Student’s paired t-test.

### Annexin-PI double staining

To assess changes in apoptotic and necrotic cell death upon the silencing of PARP10 Annexin-PI double staining was performed similarly to [22] using the FITC Annexin V/Dead Cell Apoptosis Kit (Molecular Probes, Eugene, Oregon, USA) kit according to the manufacturer’s instructions.

### ATP measurement

ATP content was measured using an ATP assay Kit (Sigma) following the manufacturer’s instructions.

### Database screening

The effect of PARP10 expression on lung, breast, colon, and ovarian cancer survival was assessed through the Kaplan-Meier plotter database (http://kmplot.com/analysis/ [23, 24]). Overall survival rates were analyzed.

### Animal experiments

All animal experiments were carried out according to the local national, EU and NIH ethical guidelines and were approved and authorized by the Institutional Animal Care and Use Committee (IACUC) at the University of Debrecen (7/2010 DE MÁB). C57/Bl6J male mice, purchased from Charles River Laboratories (Wilmington, MA, USA), had *ad libitum* access to water and food, and were kept under a 12/12 h dark-light cycle (light 7 a.m.– 7 p.m., night 7 p.m.– 7 a.m.). Mice were kept on chow diet (10 kcal% of fat) (SAFE, Augy, France). Mice were subjected to CLAMS experiment as described in [25, 26].

### Statistical analysis

Significance between groups was analyzed by paired Student’s t-test.

## Results

### Silencing of PARP10 induces mitochondrial oxidation and AMPK activity

**Fig 1:**
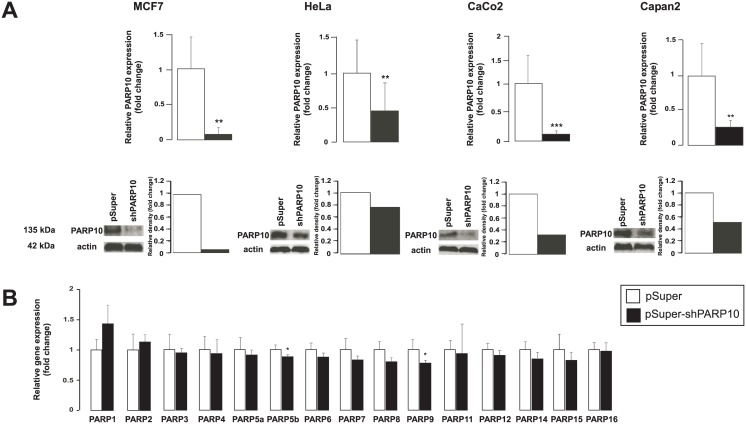
The characteristics of PARP10 silencing in MCF7, HeLa, CaCo2 and Capan2 cell lines. (A) PARP10 silencing in the indicated cell lines was assessed by RT-qPCR (n = 6/6) and Western blotting. A typical experiment is shown as average ± SD. ** and *** indicate statistically significant difference between control and transfected samples at p<0.01 and p<0.001, respectively. (B) In CaCo2 cells the specificity of the PARP10 shRNA constructs was assessed by measuring the expression of the PARP family members in RT-qPCR reactions (n = 4/4). A typical experiment is shown as average ± SD. * indicate statistically significant difference between control and transfected samples at p<0.05.

We used different, well-characterized cancer-derived cell lines in our experiments: MCF-7 as a model of breast cancer, HeLa as a model of cervix cancer, CaCo2 as a model of colorectal adenocarcinoma and Capan2 as a model for pancreas adenocarcinoma. In these models we silenced PARP10 using a mixture of two PARP10-specific pSuper constructs and using the empty pSuper construct as control. The mixture of the two PARP10 specific shRNA constructs [12] efficiently decreased the expression PARP10 in the four cell lines both at the mRNA and at the protein level (Fig 1A). The PARP10-specific shRNA proved to be specific for PARP10, none of the other members of the PARP family showed decreased expression, only slight (~10–20%) decrease was observed in the expression of PARP9 and PARP5b (TNK2) (Fig 1B).

**Fig 2:**
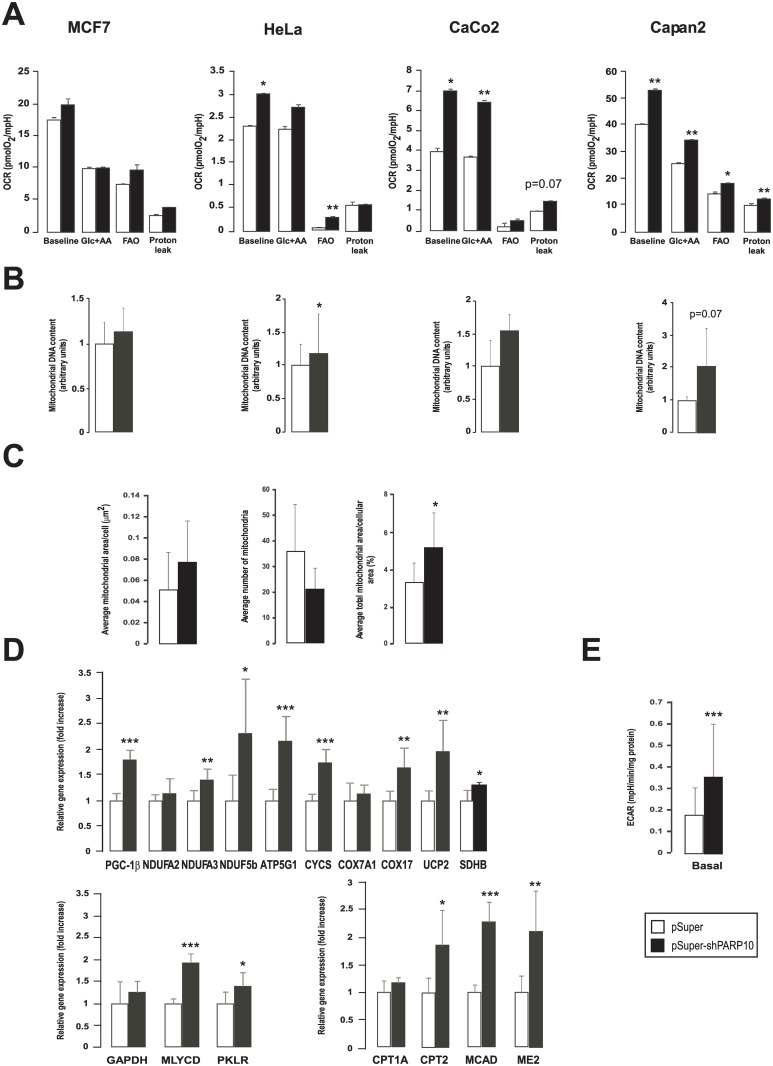
The shRNA silencing of PARP10 induces mitochondrial oxidative activity. (A) The mitochondrial oxygen consumption rate (OCR) were assayed by Seahorse XF analyzer after control shRNA or shPARP10 transfection as described in Materials and Methods (n = 23/23/23/23). A typical experiment is shown as average ± SD. * and ** indicate statistically significant difference between control and transfected cells at p<0.05 and p<0.01, respectively. (B) Mitochondrial DNA content was analyzed using qPCR in the indicated cell lines two days post transfection with control shRNA or shPARP10 (n = 3/3/3/3) as described in the Materials and Methods. Bars represent fold changes relative to control samples. All gene abbreviations are listed in the text. A typical experiment is shown as average ± SD. *, ** indicate statistically significant difference between control and transfected cells at p<0.05 and p<0.01, respectively. (C) To assess mitochondrial content and morphology electron microscopy was performed on control and PARP10-depleted CaCo2 cells followed by morphometry (n = 10/10). A typical experiment is shown as average ± SD. Representative images are provided, bar equals 2.5 μm. * indicate statistically significant difference between control and transfected cells at p<0.05. (D) The expression of genes involved in mitochondrial oxidative capacity, fatty acid oxidation and glycolysis was assessed in RT-qPCR reactions in control and PARP10-depleted CaCo2 (n = 4/4). Abbreviations are in the text. A typical experiment is shown as average ± SD. *, ** or *** indicate statistically significant difference between control and transfected cells at p<0.05 p<0.01 or p<0.001 respectively. Bars represent fold changes relative to control samples. (E) ECAR was assayed by Seahorse XF analyzer after two days transfection as described in Materials and Methods (n = 23/23/23/23). A typical experiment is shown as average ± SD. *** indicate statistically significant difference between control and transfected cells at p<0.001.

We assessed changes in mitochondrial oxidative metabolism through oximetry (Fig 2A). The silencing of PARP10 enhanced baseline OCR in Hela, CaCo2 and Capan2, with the largest effect seen in CaCo2 cells. In MCF7 cells a trend to enhanced OCR was seen but the increase was not significant. Next, we assessed changes in substrate preference for mitochondrial catabolism. Mitochondrial fatty acid oxidation was blocked by 50 μM of etomoxir, which inhibits carnitin palmitoyltransferase and substrate preference was judged as change in OCR. Fatty acid oxidation (oxygen consumption blocked by etomoxir) was enhanced in all cell lines upon PARP10 silencing, however it was statistically significant only in Hela and Capan2 cells (Fig 1B, FAO = baseline—Glc+AA). The etomoxir resistant respiration (mostly attributed to glucose and amino acid oxidation (Glc+AA)) was also enhanced by the ablation of PARP10 except for MCF7. PARP10 silencing enhanced oligomycin-resistant respiration, except in HeLa cells, that is an approximation of the proton leak through the mitochondrial inner membrane. In line with the increased mitochondrial oxidation rate, cellular mitochondrial DNA content increased in HeLa and Capan2 cells, while the increase in MCF7 and CaCo2 cells was not significant (Fig 2B).

We assessed changes in mitochondrial content by performing morphometry on electron microscopy sections. The shRNA silencing of PARP10 resulted in a decrease in the numbers mitochondria. However, the average surface areas of mitochondria and the total surface areas of mitochondria in proportion to the surface areas of the cytoplasm of the investigated cells were much higher in the PARP10-silenced cells (Fig 2C). These readouts may suggest mitochondrial biogenesis and mitochondrial fusion, both linked to enhanced mitochondrial oxidative capacity [25–30]. Improved mitochondrial oxidative capacity was also evidenced by the enhanced expression of mitochondrial genes (Nduf2a, Nduf3a, Nduf5b, peroxisome proliferator activated receptor cofactor (PGC)-1β, uncoupling protein-2 (UCP2), ATP5g1, cytochrome c (Cytc), Cox7a1, COX17, succinate dehydrogenase (SDH)), fatty acid oxidation genes (carnitin palmitoyl transferase (CPT)1, CPT2, medium-chain acyl-CoA dehydrogenase (MCAD) and malic enzyme 2 (ME2)) and genes involved in glycolysis (glyceraldehyde-3-phosphate dehydrogenase (GAPDH), malonyl-CoA decarboxylase (MYLCD), pyruvate kinase liver isoform (PKLR)) (Fig 2D). The induction of glycolytic genes was translated into enhanced glycolytic flux (Fig 2E).

**Fig 3:**
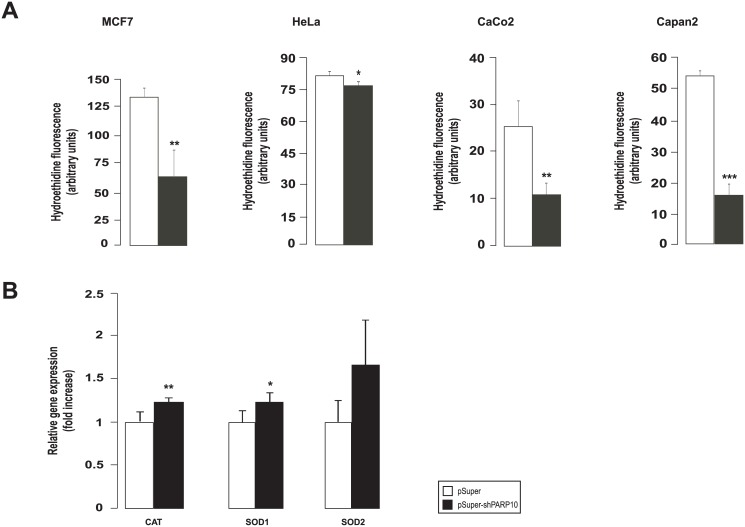
The shRNA silencing of PARP10 reduces free radical production. (A) Superoxide production was measured in the indicated cell lines using hydroethidine (HE) staining after transfecting cell twice with control shRNA or shPARP10 as described in Materials and Methods. 20,000 events collected for each sample, experiments were performed three times, results are the average of these experiments ± SEM. *, ** and *** indicate statistically significant difference between control and transfected cells at p<0.05, p<0.01 and p<0.001, respectively. (B) The expression of antioxidant genes was assessed in RT-qPCR reactions in control and PARP10-depleted CaCo2 cells (n = 4/4). A typical experiment is shown as average ± SD. *, ** indicate statistically significant difference between control and transfected cells at p<0.05 and p<0.01, respectively. Bars represent fold changes relative to control samples. Abbreviations are in the text.

We also assessed cellular reactive species production and found that cellular hydroethidine fluorescence decreased (Fig 3A) suggesting lower superoxide production in PARP10-silenced cells. These were in line with the increased the mRNA expression of catalase (CAT), superoxide dismutase (SOD)1 and SOD2 increased (Fig 3B).

**Fig 4:**
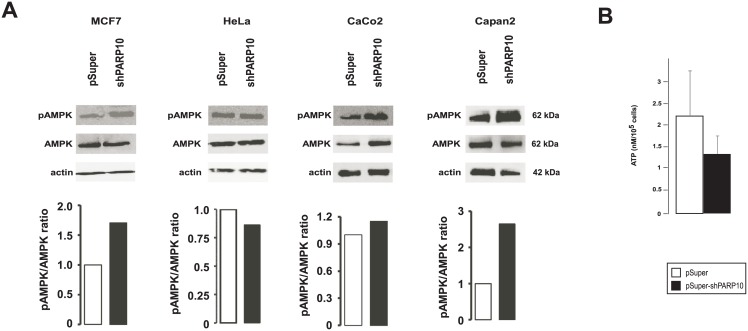
The shRNA silencing of PARP10 induces AMPK. (A) The indicated cells were transfected with pSuper or the shPARP10 constructs for 2 days then cells were harvested and Western blot was performed. AMPK activity was determined by Western blot analysis of AMPK phosphorylation. The number of biological replicates for the AMPK blots in MCF7 is 3, Hela is 4, For CaCo2 is 3 and for Capan is 3; for the pAMPK blots in MCF7 is 4, Hela is 2, For CaCo2 is 3 and for Capan is 2. Actin was used as a loading control. Western blots were subjected to densitometry using the ImageJ software, a typical results is shown. On the pAMPK blots for HeLa and MCF7 brightness and contrast was adjusted. Abbreviations are in the text. (B) In control and PARP10-depleted CaCo2 (n = 4/4) ATP levels were determined. A typical experiment is shown as average ± SD.

We aimed to identify the molecular background of the enhancement of mitochondrial oxidative capacity. As PARP10 was described to interfere with energy/metabolite sensor pathways (e.g. GSK3β [12] or c-MYC [11]) we also assessed a typical energy sensor pathway that converges on the mitochondria, AMP-activated kinase (AMPK) [31]. AMPK acts as a heterotrimer (α, β and γ subunits) that is activated by cellular energy stress in response to increased in AMP/ATP ratio. Upon activation AMPK inhibits anabolism and stimulates catabolism by phosphorylating a plethora of target proteins [31]. Upon silencing of PARP10 the phosphorylation of the AMPKα subunit increased except for Hela cells (Fig 4A), suggesting enhanced AMPK activation in MCF7, CaCo2 and Capan cell lines, although the change in phosphorylation varied between cell lines. Furthermore, silencing of PARP10 reduced ATP levels that is a likely trigger for AMPK (Fig 4B).

### Silencing of PARP10 exerts anti-Warburg features in tumors of the gastrointestinal tract

As next step, we sought (patho)physiological processes where PARP10-mediated in oxidative metabolism may play role. Tumor cells are characterized by a complex rearrangement of metabolic processes that is referred to as the Warburg effect [8, 32]. Although, the biochemical changes that underlie this are diverse, a commonly occurring feature is the suppression of mitochondrial oxidative capacity [8]. It is also of note that the Warburg effect can be reverted by enhancing the mitochondrial oxidative metabolism, which leads to a slowdown of cell proliferation in susceptible tumors [8] suggesting that the silencing of PARP10 through inducing mitochondrial catabolism may have anti-proliferative effects.

**Fig 5:**
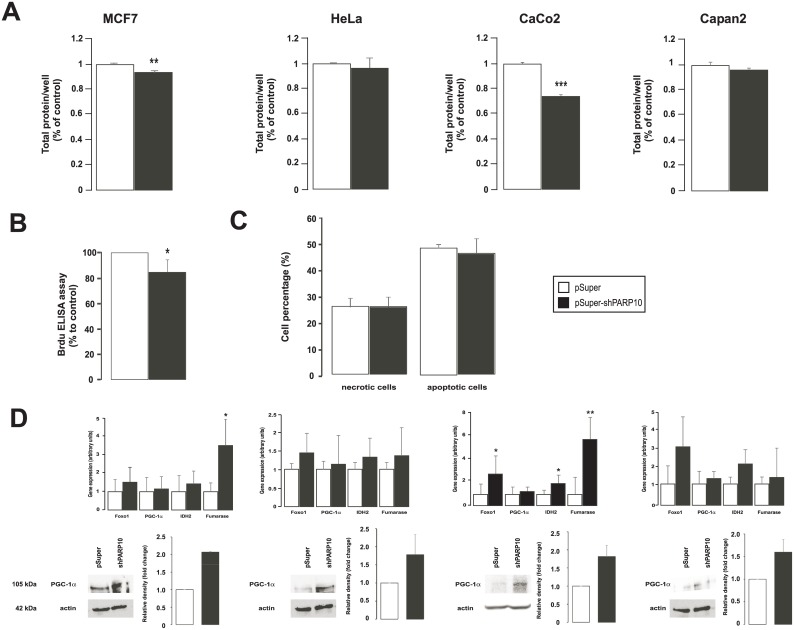
The shRNA silencing of PARP10 reduces the proliferation of MCF7 and CaCo2 cells. (A) Cell proliferation was determined by sulphorhodamine B assay upon ablation of PARP10 in the cell lines indicated as described in Materials and Methods (n = 3). Experiments were performed three times, results are the average of these experiments ± SEM. (B) Cell proliferation was assessed in CaCo2 cells (n = 8/8) by measuring BRDU incorporation after two days of control or PARP10 shRNA transfection. A typical experiment is shown as acverage ± SD. (C) The ratio of apoptotic and necrotic cells were determined in CaCo2 cells (n = 3/3) transfected with control of PARP10 shRNA for 2 days. A typical experiment is shown. *, ** and *** indicate statistically significant difference between control and transfected cells at p<0.05, p<0.01 and p<0.001, respectively. (D) The expression of the genes indicated was determined in RT-qPCR assays (n = 3, average ± SEM). In the same cells PGC-1α protein levels were determined by Western blotting. The number of the biological replicates for MCF7 is 2, for Hela is 2, for CaCo is 2 and for Capan is 3. On the bar charts the average of the results and SEM is shown.

We assessed cellular proliferation upon the ablation of PARP10 in the four tumor cell lines. The silencing of PARP10 in MCF7 and in CaCo2 resulted in a significant reduction of cellular proliferation (Fig 5A) as shown in sulphorhodamine B assays. The biggest change was observed in the case of CaCo2 cells. We verified reduced proliferation in BRDU-incorporation assays in CaCo2 cells that showed a similar pattern as the SRB assay (Fig 5B). Importantly, we have not detected changes in the rate of necrosis and apoptosis (Fig 5C) that underlined the cytostatic, but not cytotoxic property of PARP10 silencing. Next, we assessed the expression of genes involved in Warburg-type metabolism in different tumors. These genes are often mutated or underexpressed in tumors [33–38]. Although, in all cell lines the silencing of PARP10 increased the mRNA and protein expression of these genes to a certain extent, increases were statistically significant only in CaCo2 cells (Fig 5D). This coincided well with the observed stimulation of mitochondrial oxidative capacity and reduction of cell proliferation.

**Fig 6:**
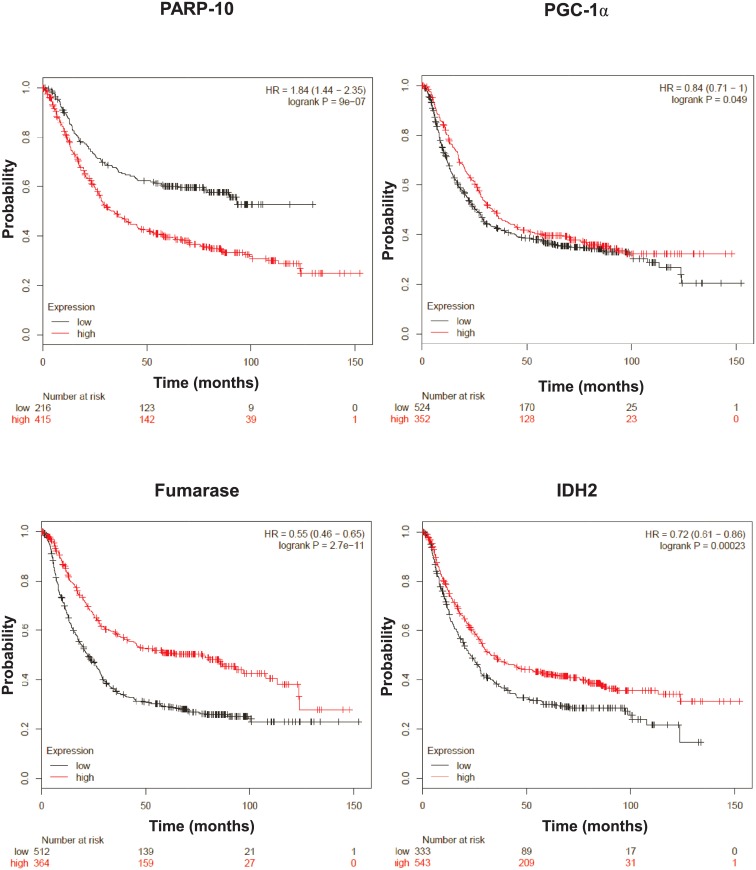
Lower expression of PARP10 and higher expression of enzymes involved in oxidative phosphorylation confer protection against gastric cancer. kmplot.com, a freely accessible database was screened for the genes indicated in patients suffering from gastric cancer. Overall survival rates were analyzed and all patients are depicted.

Subsequently, we assessed whether these findings may have human implications. We assessed an online database (kmplot.com) that collects gene expression profiles human tumors and survival the corresponding cancer patients. Using this database we found that low PARP10 expression, as well as, higher expression of the previously described marker genes (PGC-1α, fumarase and isocitrate dehydrogenase-2 (IDH2)) provides longer survival in gastric cancer (Fig 6).

### PARP10 expression in metabolic tissues correlates with fasting

**Fig 7:**
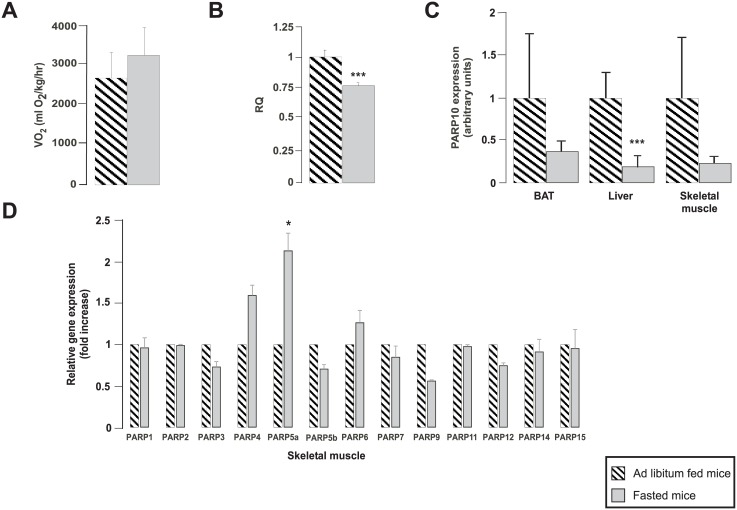
PARP10 expression changes inversely as mitochondrial oxidative capacity in mice. C57/Bl6 male mice were subjected to 16 hours of fasting or received ad libitum food (n = 4/4, 3 months of age, average ± SEM). (A) The oxygen consumption and (B) the RQ were determined in these animals in indirect calorymetry experiments. (C) After dissection, the expression of PARP10 mRNA was determined by RT-qPCR in the brown adipose tissue (BAT), liver and skeletal muscle. (D) The expression of the members of the PARP family was determined in skeletal muscle using RT-qPCR reactions. Data is represented as average ± SEM. *, *** indicate statistically significant difference between control and transfected cells at p<0.05 or p<0.001, respectively.

Besides Warburg metabolism another physiological stress that induces mitochondrial catabolism is fasting [9]. Under fasting, anabolic processes are shut down, while catabolism is enhanced to support survival. A key factor for adaptation to starving is the activation of mitochondrial oxidative capacity [9] that prompted us to assess changes in PARP10 expression in response to fasting in metabolic tissues. Fasting and ad libitum (AL)-fed mice were subjected to an indirect calorimetry experiment. Fasted mice were deprived of food for 16 hours that led to an increase in oxygen consumption (Fig 7A), indicating mitochondrial activation and reduction in respiratory quotient (RQ) suggesting enhanced fatty acid oxidation (a process that frequently involves mitochondrial activation) (Fig 7B). In line with enhanced mitochondrial oxidative capacity PARP10 expression was reduced significantly in the liver and PARP10 expression in the brown adipose tissue and skeletal muscle showed a similar trend too (Fig 7C). We assessed whether the expression of other PARPs also changes upon fasting. Interestingly, the expression of PARP4, PARP5a and PARP6 were induced, while the mRNA expression of PARP3, PARP5b, PARP9 and PARP12 were reduced upon fasting (Fig 7D).

**Fig 8:**
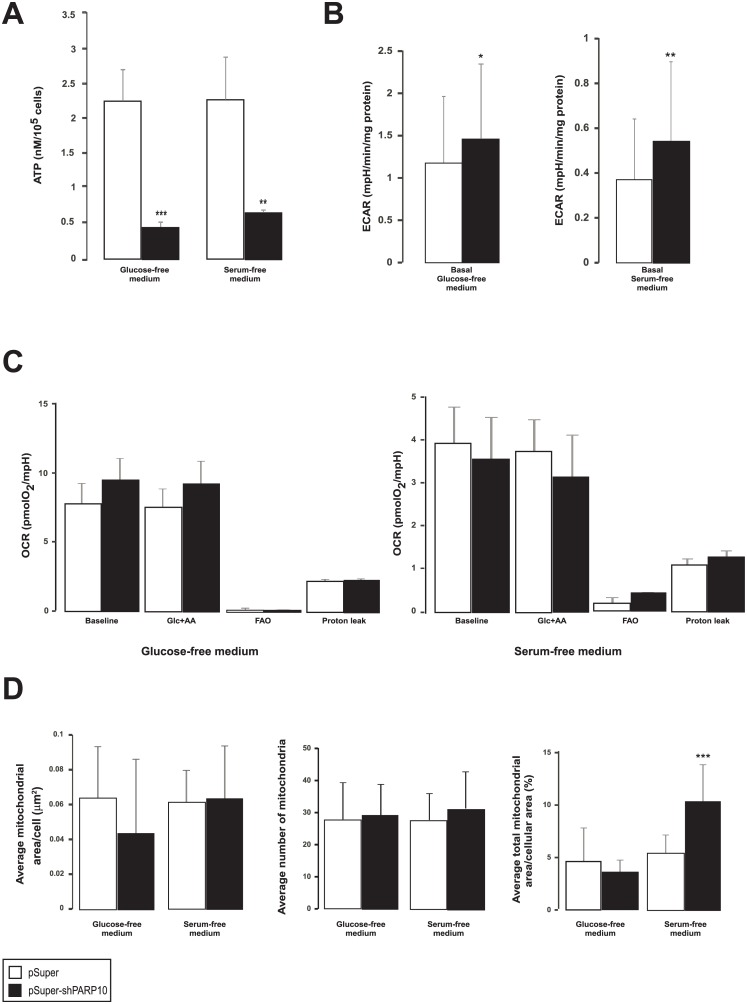
PARP10-induced mitochondrial biogenesis is abolished upon glucose or serum fasting. (A) CaCo2 cells (n = 4/4) were transfected with control or PARP10 shRNA for two days then ATP levels were determined. A typical experiment is shown as average ± SD. (B) ECAR was assayed by Seahorse XF analyzer in CaCo2 cells after two days transfection as described in Materials and Methods (n = 23/23/23/23). A typical experiment is shown as average ± SD. (C) The mitochondrial oxygen consumption rate (OCR) was assayed by Seahorse XF analyzer after two days transfection as described in Materials and Methods (n = 23/23/23/23). A typical experiment is shown as average ± SD. (D) To assess mitochondrial content and morphology electron microscopy was performed on control and PARP10-depleted CaCo2 cells (n = n = 8/13/8/18). A typical experiment is shown as average ± SD. **, *** indicate statistically significant difference between control and transfected cells at p<0.011 and p<0.001, respectively.

We performed additional assays to gain better insight into the PARP10-driven metabolic processes upon serum or glucose fasting. ATP levels were further reduced upon glucose or serum fasting as compared to controls (Figs 4C vs. 8A) suggesting aggravated energy stress. In serum and glucose-fasted cells ECAR vales were higher in the PARP10-silenced cells (Fig 8B), however, to a way lower extent than in non-fasted cells (Fig 2E). In a similar fashion to these, increases in OCR values upon PARP10 silencing were lower upon glucose fasting, while in serum-fasted cells OCR values were even lower in shPARP10 cells than in control cells (Fig 8C). The morphological changes suggesting mitochondrial biogenesis in non-fasted cells (Fig 2C) were also abolished by glucose or serum-fasting (Figs 2C vs. 8D).

## Discussion

In the present study we identify PARP10 as a metabolic regulator. The ablation of PARP10 enhanced mitochondrial oxidative capacity and glycolysis, furthermore, induced fatty acid oxidation in our experiments. As a possible molecular explanation to these observations we showed that changes in PARP10 expression inversely correlated with the activity of AMPK and with the expression of key mitochondrial genes, among them, PGC-1α.

What could be the link between PARP10, AMPK and PGC-1α? AMPK is activated upon cellular energetic stress marked by decreases in ATP and/or increases in AMP levels [39]. Our study revealed that ATP levels drop upon the silencing of PARP10 and therefore it is a possible explanation that the depletion of PARP10 declutches cellular energy stress that in turn activate AMPK. Continuing along this hypothesis, AMPK activation, provoked by energy stress, is responsible for the upregulation of catabolism, nevertheless, the actual steps how PARP10 achieves that are unknown warranting further investigations in this direction. Interestingly, when cells are further stressed by glucose or serum deprivation we were able to further depress ATP levels, but the signs of PARP10-induced mitochondrial biogenesis were generally reduced pointing out that fasting-induced and PARP10-induced energy stress are not additive. It is also of note that another ADP-ribosyltransferase, PARP-1, was reported to physically interact with AMPK [40] and it seems that both enzymes can cross activate each other. AMPK phosphorylates PARP-1 [40], while PAR degradation yields AMP [41] that stimulates AMPK [5].

We have also observed increases in the expression of PGC-1α in cells where PARP10 was silenced. PGC-1α is also a key metabolic regulator, increases in PGC-1α expression or activity supports mitochondrial biogenesis through facilitating the *de novo* expression of mitochondrial genes [30, 42, 43], furthermore, mutations or decrease in the expression of PGC-1α blunt mitochondrial oxidative metabolism and hence at the level of physiology may predispose to type II diabetes [44, 45]. The activation of other PARP enzymes, such as PARP1, PARP2 and TiPARP, were already shown to inhibit PGC-1α activity [25, 26, 46–48]. How PARP10 can influence PGC-1α expression is unknown, however, it should be mentioned that AMPK activation through the consequent induction of SIRT1 can positively regulate PGC-1α [29, 49] that may contribute to PGC-1α activation upon the silencing of PARP10.

PARP10-mediated AMPK activation can modulate metabolic adaptation in cancer cells and in different metabolic tissues upon fasting. AMPK activation in those cancers that rely on Warburg metabolism, like breast or colorectal cancer, counteracts cancer growth [38, 50]. Although, we detected AMPK activation in all cell lines we investigated, only two responded to PARP10 ablation by reduced proliferation, the breast cancer cell line MCF-7 and the colorectal carcinoma cell line CaCo2, however, the other two cell lines also displayed a tendency towards reduced proliferation. It seems therefore that other, cell/tissue-specific signaling pathways also contribute to reduced proliferation capacity upon the silencing of PARP10. It is also noteworthy that PARP10 is an interacting partner of c-Myc and through that, PARP10 protects against the transformation of embryonic fibroblasts [16]. Our data support that PARP10 is necessary for cellular proliferation, however add that the effects of PARP10 appears to be tissue/cancer dependent.

Changes in mitochondrial oxidative function plays a key role in fasting. Fasting induces mitochondrial oxidative activity that frequently correlates with fatty acid oxidation in metabolic tissues [9]. PARP10 expression correlated inversely with mitochondrial oxidative capacity (marked by increases in oxygen consumption) in the liver with similar tendency in the brown adipose tissue and skeletal muscle, all key tissues of energy expenditure, fatty acid and glucose homeostasis [9]. Taken together in cancer and metabolic models PARP10 expression was regulated inversely as mitochondrial oxidative capacity. On broader perspective, these data also suggest that lower PARP10 expression may bring about a negative energy balance through enhancing mitochondrial biogenesis and energy expenditure *in vivo*.

Another important observation in our studies was that low PARP10 expression associated with increases in fatty acid oxidation. Namely, increases in the expression of fatty acid oxidation genes or etomoxir-sensitive respiration and low RQ values in fasting that is associated with low PARP10 expression. In fact, AMPK activation has been shown to enhance fatty acid oxidation [31, 51, 52] that makes it tempting to speculate that PARP10-induced AMPK activation may stand behind enhanced fatty acid oxidation. A genome wide association study associated PARP10 to apolipoprotein B expression [18] further pointing towards a role for PARP10 in lipid homeostasis. It is of note that PARP1 and PARP2 are both associated with lipid metabolism [3]. PARP1 and PARP2 are involved in the regulation of organismal and tissular triglyceride and fatty acid homeostasis, furthermore cholesterol and lipoprotein metabolism [25, 26, 53–62]. Our study nominate PARP10 as a PARP that is involved in the regulation of fatty acid degradation.

On a broader perspective, other cell types and other processes can be under the influence of the interaction between PARP10 and AMPK/mitochondria (e.g. beige cells [63–65], macropahges [66]) warranting further studies towards the understanding of the role of PARP10 in metabolic regulation.
